# The Role of 1‐Methylcyclopropene in the regulation of ethylene biosynthesis and ethylene receptor gene expression in *Mangifera indica* L. (Mango Fruit)

**DOI:** 10.1002/fsn3.1417

**Published:** 2020-01-20

**Authors:** Li Li, Liang Shuai, Jian Sun, Changbao Li, Ping Yi, Zhugui Zhou, Xuemei He, Dongning Ling, Jinfeng Sheng, Kin‐Weng Kong, Fengjin Zheng, Jiemin Li, Guoming Liu, Ming Xin, Zhichun Li, Yayuan Tang

**Affiliations:** ^1^ Agro‐food Science and Technology Research Institute Guangxi Academy of Agricultural Sciences Nanning China; ^2^ Guangxi Key Laboratory of Fruits and Vegetables Storage‐processing Technology Nanning China; ^3^ Department of Molecular Medicine Faculty of Medicine University of Malaya Kuala Lumpur Malaysia

**Keywords:** 1‐methylcyclopropene, cloning and expression, ethylene biosynthesis, ethylene receptor genes, *Mangifera indica* L., regulation

## Abstract

Mango (*Mangifera indica* L.) is respiratory climacteric fruit that ripens and decomposes quickly following their harvest. 1‐methylcyclopropene (1‐MCP) is known to affect the ripening of fruit, delaying the decay of mango stored under ambient conditions. The objective of this study was to clarify the role of 1‐MCP in the regulation of ethylene biosynthesis and ethylene receptor gene expression in mango. 1‐MCP significantly inhibited the 1‐aminocyclopropane‐1‐carboxylic acid (ACC) content. The activity of ACC oxidase (ACO) increased on days 6, 8, and 10 of storage, whereas delayed ACC synthase (ACS) activity increased after day 4. The two homologous ethylene receptor genes, *ETR1* and *ERS1* (i.e., *MiETR1* and *MiERS1*), were obtained and deposited in GenBank^®^ (National Center for Biotechnology Information‐National Institutes of Health [NCBI‐NIH]) (KY002681 and KY002682). The *MiETR1* coding sequence was 2,220 bp and encoded 739 amino acids (aa). The *MiERS1* coding sequence was 1,890 bp and encoded 629 aa, similar to *ERS1* in other fruit. The tertiary structures of MiETR1 and MiERS1 were also predicted. MiERS1 lacks a receiver domain and shares a low homology with MiETR1 (44%). The expression of *MiETR1* and *MiERS1* mRNA was upregulated as the storage duration extended and reached the peak expression on day 6. Treatment with 1‐MCP significantly reduced the expression of *MiETR1* on days 4, 6, and 10 and inhibited the expression of *MiETR1* on days 2, 4, 6, and 10. These results indicated that MiETR1 and MiERS1 had important functions in ethylene signal transduction. Treatment with 1‐MCP might effectively prevent the biosynthesis of ethylene, as well as ethylene‐induced ripening and senescence. This study presents an innovative method for prolonging the storage life of mango after their harvest through the regulation of *MiETR1* and *MiERS1* expression.

## Graphical abstract

In this study, role of 1‐Methylcyclopropene in the regulation of ethylene biosynthesis and ethylene receptor gene expression in mango fruits were studied. The work reports the role of ethylene receptor family members MiETR1 and MiERS1 in response to senescence related signaling in postharvest mango fruit.

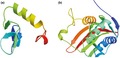

## 1 INTRODUCTION

Ethylene is a gaseous plant hormone that is involved in plant growth and development, including fruit ripening, fruit abscission, leaf senescence, seed germination, and organogenesis (Bleecker & Kende, 2000; Trujillo‐Moya & Gisbert, 2012). It also regulates various stress responses in plants, including water deficits, mechanical wounds, and pathogen attacks (Jiang & Fu, 2000; Martínez, Gómez, & Gómez‐Lim, 2001). Ethylene is generated following the catalysis of 1‐aminocyclopropane‐1‐carboxylic acid (ACC) by an enzyme called ACC oxidase (ACO). Pathak, Asif, Dhawan, Srivastava, and Nath (2003) reported that ACO was constitutively expressed at low levels and was induced during banana fruit ripening. In addition, the transcriptional activity of the ACC synthase (ACS) gene may regulate the synthesis of ethylene.

The ethylene receptor is an upstream element that is encoded by a multigene family, playing a negative regulatory role in the ethylene signal transduction pathway. The receptor proteins encoded by each gene have different structures and levels of expression. Five *ETR1*‐like genes, which are *ETR1* (Chang, Kwok, Bleecker, & Meyerowitz, 1993), *ERS1* (Hua, Chang, Sun, & Meyerowitz, 1995), *ETR2* (Sakai et al., 1998), *EIN4*, and *ERS2* (Hua et al., 1998), are identified in *Arabidopsis* plants. Although *ETR1* and *ERS1* are ubiquitously expressed in *Arabidopsis* plants, they demonstrate distinct expression patterns in different tissues (Hua et al., 1998). The overall modular structures of ethylene receptors are similar, with a transmembrane domain near the N‐terminus, accompanied by an undetermined functional GAF domain and domains for signal output in the C‐terminus (Shakeel, Wang, Binder, & Schaller, 2013). Three putative membrane‐spanning subdomains were found in the N‐terminal hydrophobic region of the ETR1 protein, which may assist in forming the ethylene‐binding site. The C‐terminus of ETR1 contains histidine protein kinase and receiving domains and may participate in delivering the ethylene signal. Although the protein encoded by the ERS1 gene has sequence similarities to the histidine kinase domain and the N‐terminus of ETR1, it lacks a receiving domain. In a two‐hybrid in vitro binding experiment, the C‐terminal regions of both ETR1 and ERS1 were shown to interact with the rapidly accelerated fibrosarcoma kinase‐like protein known as constitutive triple response 1 (CTR1) and to negatively regulate the transduction pathway of ethylene (Kuroda, Hirose, Shiraishi, Davies, & Abe, 2004).

It has been demonstrated that the ethylene action inhibitor 1‐methylcyclopropene (1‐MCP) affects plant senescence via irreversibly binding to the ethylene‐binding receptor, thereby prohibiting the ethylene signal in the transduction pathway. Amornputti, Ketsa, and Doorn (2016) reported that 1‐MCP inhibited the production of ethylene in fruit, which was correlated with the ACC content and the activity of ACO and ACS. Rasori, Ruperti, Bonghi, Tonutti, and Ramina (2002) reported that 1‐MCP delayed peach fruit maturation by estimating the fruit firmness and release of ethylene. In addition, 1‐MCP may downregulate ERS1 without affecting the transcription of ETR1 (Rasori et al., 2002). Li, Qiao, Tong, Zhou, and Zhang (2010) showed that 1‐MCP upregulated the expression of the *ETR3* gene in pear fruit 0 d–9 d after harvest and downregulated the expression of *ERS2* 6 d–15 d after harvest. Karakurt, Tonguç, and Ünlü (2014) confirmed that 1‐MCP markedly decreased the expression levels of the *ETR1*, *ERS1*, and *ETR2* genes in watermelons. Moreover, 1‐MCP has been broadly used to delay ripening and senescence in plants and to consequently extend the storage life of climacteric fruit.

Mango is a fruit with a great market value. However, the storage life of mango is short (Sherman et al., 2015). Although mangos are harvested in the mature‐green stage, they ripen quickly and often decay while being traded (Lalel, Singh, & Tan, 2003). The short postharvest life of mango limits their consumption (Salinas‐Roca et al., 2018). Mango naturally produces ethylene; thus, controlling ethylene production is an appealing strategy to extend their storage life. Ascertaining the function of the ethylene receptors may assist in elucidating the regulatory mechanism of ethylene in postharvest mango. Unfortunately, the evidence on the expression of the *ETR1* and *ERS1* receptors in mango is currently limited. The shared common function and specific roles of *ETR1* and *ERS1* remain unknown. The role of 1‐MCP in the regulation of ethylene receptor gene expression has not been investigated in mango. Therefore, it is necessary to elucidate the mechanisms of ethylene receptor gene expression in response to treatment with 1‐MCP at the molecular level.

Regulation of ethylene biosynthesis has an impact on ripening and senescence of mango fruit. The aim of this study was to clarify the role of 1‐MCP in the regulation of ethylene biosynthesis and ethylene receptor gene expression in mango after their harvest. In particular, the role of 1‐MCP in the regulation of *MiETR1* and *MiERS1* expression and fruit storage life was investigated. The complementary DNA (cDNA) of *MiETR1* and *MiERS1* was amplified, and analyses of the structures and sequences of the two genes were conducted. The biochemical characteristics of the *MiETR1* and *MiERS1* isoforms were studied in order to determine specific roles in ethylene signaling and investigate biochemical similarities and differences. In addition, the role of 1‐MCP in the regulation of the physiological characteristics and ethylene biosynthesis in mango was investigated in order to decipher the underlying mechanism of 1‐MCP in delaying senescence. This study presents an innovative method for prolonging the storage life of mango after their harvest through the regulation of the expression of *MiETR1* and *MiERS1*.

## 2 MATERIALS AND METHODS

### 2.1 Plant materials and treatments

Experimental cultivar “Tainong 1” mango fruit (*Mangifera indica* L.) were harvested from an orchard in Tianyang, Baise City, Guangxi Province, China), in July 2016. Fruits with a similar shape, size, and physiological maturity were selected as experimental materials. These fruits were transported to the Guangxi Key Laboratory of Fruits and Vegetables Storage‐Processing Technology (Nanning, China) immediately after harvest and were randomly classified into two groups (60 fruits per group). 1‐MCP (Beijing PLM Biosciences Co.) was purchased in a powder form and dissolved in sterile distilled water at a final concentration of 1 μl/L. One group was placed in polyethylene bags (0.03 mm thick) containing 1 μl/L 1‐MCP. The other group was treated with the same volume of sterile water only (control group). The fruits were treated with 1‐MCP for 24 hr at 25°C and subsequently stored at 25°C and were then collected every two days, frozen in liquid nitrogen, and stored at −80°C until analysis. Three replicates per analysis were used for all measurements.

### 2.2 Evaluation of firmness, TSS,and TA of mango fruit

Firmness in the 1‐MCP‐treated and control groups was measured using a handheld FT‐327 penetrometer (UC Fruit Firmness Tester, Milano, Italy) with an 8 mm diameter probe. Following the removal of a small piece of fruit skin, the firmness from three slices extracted from three different parts of the mango was recorded (means are presented in newtons [N]). The total soluble solid (TSS) content of the fruit was examined on the basis of the AOAC method (AOAC, 2000) using a handheld refractometer (ATAGO, Tokyo, Japan). Mango fruit juice was titrated with 0.1 N NaOH to pH 8.2 in order to determine the titratable acidity (TA) using an automatic titrator (TitroLine Easy, Schott, Mainz, Germany).

### 2.3 Rates of ethylene production and respiration

Postharvest fruits from the 1‐MCP‐treated and control groups were placed in pure N_2_ and subsequently stored at 20°C with 90% relative humidity. Three fruits were placed in a 4.2  L airproof glass jar for 2 hr at 25°C to determine the rates of ethylene production at different storage stages. A headspace gas sample (1 ml) was collected from each jar and analyzed using a GC‐2014C gas chromatograph (Shimadzu, Kyoto, Japan). Subsequently, a thermal conductivity detector (Shimadzu TCD‐2014) with Porapak N column was used to detect the concentration of carbon dioxide in the samples. The levels of ethylene were determined using a flame ionization detector and an OV‐17 capillary column (Zhonghuida Co.) (An, Zhang, Lu, & Zhang, 2006). The rates of ethylene production and respiration are presented on a FW basis that mention above.

### 2.4 Determination of the ACC content and activity of the ACO and ACS

A total of 10 g of mango fruit pulp was collected from the 1‐MCP‐treated and control groups and homogenized in 25 ml of 80% (v/v) ethanol at 4°C. The homogenates were centrifuged for 10 min at 10,000*g*, and the supernatant ethanol was removed using a SpeedVac^™^ concentrator (Thermo Fisher Scientific, Waltham). The remaining pellets were resuspended in distilled water, and the ACC content was determined on the basis of the method described by Boller, Herner, and Kende (1979) and the protocol by Lizada and Yang (1979). The extraction of ACO and ACS was conducted, and their activities were determined according to the method described by Zheng, Nakatsuka, Taira, and Itamura (2005).

### 2.5 RNA extraction

Total RNA was isolated from 100 mg of fresh mango tissue pulverized using liquid nitrogen. RNA was isolated on the basis of the method described by López‐Gómez and Gómez‐Lim (1992). An RNAprep Pure Kit for plants (Tiangen Co., Beijing, China) was utilized according to the manufacturer's instructions, followed by treatment with RNase‐free DNase (Takara Biotechnology) in order to purify the samples. The RNA quantity was assessed using a NanoDrop^®^ ND‐1000 UV‐Vis spectrophotometer (NanoDrop Technologies Inc.) at 260 nm. A 2,100 Bioanalyzer system (Agilent Technologies) was used to evaluate RNA integrity.

### 2.6 Full‐length cDNA cloning of *MiETR1* and *MiERS1*

Full‐length cDNA sequences encoding *MiETR1* and *MiERS1* were acquired from the combination of RT‐PCR and rapid amplification of cDNA ends (RACE) cloning. RNA (10 µg) was used to synthesize first‐strand cDNA. All primers and reagents in cloning were purchased from the PowerScript^™^ MMLV reverse transcriptase (Clontech). The degenerate primers of the *MiETR1* conservative region, PD‐cons1 (5′‐GCCCTGATGCTGGTGCAYATHATHCC‐3′) and PD‐cons1 (5′‐GGTTCATCACGGCCARRAARTCRTT‐3′), were designed according to the homologous sequence of *Arabidopsis thaliana* (accession no. NP_176808.3). The specific primers of the *MiERS1* conservative region, PD‐cons1 (5′‐ATGCCTTCAAGAACTGGTATCAC‐3′) and PD‐cons1 (5′‐CAAGTCCTGCTCCACCTGTA‐3′), were also designed on the basis of the homologous sequence of *Arabidopsis thaliana* (accession no. NP_181626.1). The PCR conditions were as follows: 94°C for 4 min; 94°C for 30 s, 53°C for 30 s, and 72°C for 90 s following 35 cycles; and 72°C for 6 min. The PCR products were used for isolation, cloning, and sequencing (Invitrogen). The 5′‐ and 3′‐RACE fragments were cloned using the SMART^™^ RACE cDNA Amplification Kit (Clontech) according to the manufacturer's instructions. The cDNA was directly utilized in 5′‐ and 3′‐RACE PCR, whereas second‐strand synthesis and adaptor ligation were not required. The 5′‐ and 3′‐RACE PCR specific primer pairs of *MiETR1* (GSP1: 5′‐TCGTGTTCCACTCCTGCATCTCTCAA‐3′, GSP2: 5′‐AAACAGCCATTCATGCTCGCAAC‐3′) were designed as the forward primer according to the sequence of the conservative region of *MiETR1*. The 5′‐ and 3′‐RACE PCR specific primer pairs of *MiERS1* (GSP1: 5′‐TTGGCTCGACAAGAGGCAGAGAAGGCAATCCAT‐3′, GSP2: 5′‐CGCCAGATTCACCAACATACTCAATT‐3′) were designed as the forward primer according to the sequence of the conservative region of *MiERS1*. The conditions of RACE PCR were as follows: 94°C for 3 min; 94°C for 30 s, 72°C for 3 min following 5 cycles; 94°C for 30 s, 70°C for 3 min following five cycles; 94°C for 30 s, 68°C for 3 min following 25 cycles; and 68°C for 3 min. The products of 5′‐ and 3′‐RACE PCR were purified and cloned into a pMD18‐T vector (Takara Bio) for sequencing (You et al., 2014). The full‐length cDNA sequences of *MiETR1* and *MiERS1* were acquired according to the sequencing results of the conservative region and the 5′‐ and 3′‐RACE products.

### 2.7 Sequence and bioinformatic analysis

Sequence alignment, translation of open reading frame (ORF), and calculation of the molecular mass of the predicted proteins were conducted using the DNAMAN v. 6.0.3.99 software and upload to the server of NCBI‐NIH (http://www.ncbi.nlm.nih.gov/blast/Blast.cgi). The putative domains of *MiETR1* and *MiERS1* were predicted using the SWISS‐MODEL (http://swissmodel.expasy.org/) with first approach and project (optimize) modes with default value. The Swiss‐PdbViewer (version 4.01, Swiss Institute of Bioinformatics, Geneva, Switzerland) was used for the visualization of structures. ClustalX v.1.81 (://www.clustal.org/) was used to align the amino acid sequences on the basis of the default settings and then refined through visual inspection. Subsequently, the minimum evolution method of the MEGA software (version 4.0, Megasoftware) was used to generate a phylogenetic tree based on the result of the alignment. Poisson's correction metric and pairwise deletion option were used for the analysis. The reliability of the tree branches was inspected by bootstrap statistical method, which created from 1,000 replicates.

### 2.8 Real‐time quantitative PCR analysis

The cDNA of postharvest mango stored for various lengths of time was obtained as described earlier in this article. These cDNA samples were used to conduct a quantitative RT‐PCR (RT‐qPCR) assay. The sequences designed for the *MiETR1* gene‐specific primers were as follows: MiETR1F (5′‐CCTACAACTTCAACTCGGAACTT‐3′) and MiETR1R (5′‐TTCATCACCAACAGCATACTCAG‐3′). The sequences designed for the *MiERS1* gene‐specific primers were as follows: MiERS1F (5′‐GTATTCTGCCACAAGACATTCCA‐3′) and MiERS1R (5′‐TCAAGACCTTCACTCTCAATCCA‐3′). The mango actin gene was used as an internal control with the following primers: MiactF (5′‐GTGGCTGTTAACGATCCCTT‐3′) and MiactR (5′‐GTGACTGGCTTCTCATCGAA‐3′). The synthesized cDNA was amplified as described earlier. RT‐qPCR was performed using the UltraSYBR Mixture (CWbio Co. Ltd., Beijing, China) in a SmartCycler^®^ system (Cepheid). Relative gene expression was calculated using the comparative Ct method (ΔΔCt) (Livak & Schmittgen, 2001). A seriated template dilution experiment was conducted using the quantization method, and a calibration curve was created. Agarose gel electrophoresis was used to determine the PCR products. The size of the expected amplicon was 130 bp.

### 2.9 Statistical analysis

All experiments were performed in triplicate (*n* = 3) with a completely randomized design. All data are presented as mean ± standard error (SE). Fisher's least significant difference test was performed using one‐way analysis of variance by SPSS software (version 13.0, IBM, New York, USA) with a significant value of *p* < .05.

## 3 RESULT

### 3.1 Effects of treatment with 1‐MCP on firmness, TSS, and TA of mango fruit

**Figure 1:**
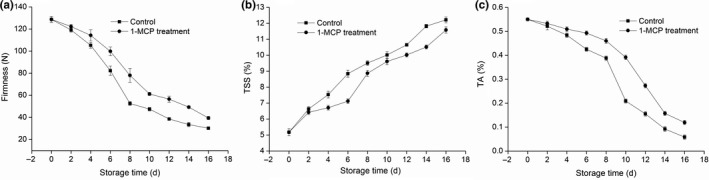
Effects of 1‐MCP on firmness (a), TSS (b), and TA (c) of mango fruit at 25°C during sixteen days. Results represent the mean ± standard error (SE)

A decreasing trend in the firmness of mango was observed during ambient storage in both 1‐MCP‐treated and control groups (Figure 1a). However, after four days of storage, loss of firmness was significantly delayed (*p* < .05) in the 1‐MCP‐treated group compared with that observed in the control group. The TSS content in the pulp tissues of both groups was shown to increase as the storage duration extended (Figure 1b). However, the TSS content in the 1‐MCP‐treated group was significantly lower than that recorded in the control group, especially on days 4, 6, and 14 of storage (all *p* < .05). The TA content in the pulp tissues of both groups decreased during the 16‐day storage. However, the TA content in the 1‐MCP‐treated group decreased more slowly than that observed in the control group after four days of storage (Figure 1c). These results revealed that treatment with 1‐MCP is effective in retaining the firmness of mango stored under ambient conditions.

### 3.2 Effects of treatment with 1‐MCP on the rates of ethylene production, respiration, and biosynthesis

**Figure 2:**
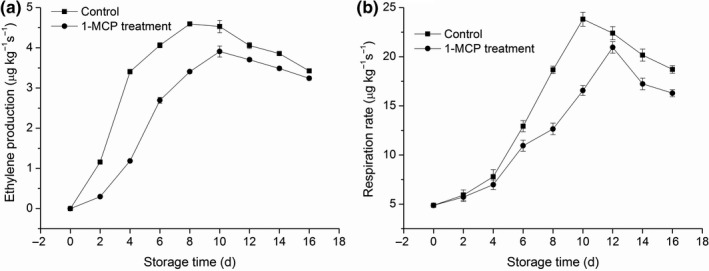
Effects of 1‐MCP treatment on ethylene production (a) and respiration rate (b) in mango fruit at 25°C during sixteen days. Results represent the mean ± standard error (SE)

Ethylene is naturally produced in postharvest mango and quickly leads to overripening. Initially, the rates of ethylene production in both groups showed an increasing trend, followed by a slow decline. Treatment with 1‐MCP significantly prevented the production of ethylene after two days of storage (*p* < .05). Production of ethylene in the 1‐MCP‐treated group peaked on day 10 of storage, two days later compared with the control group. These results indicated that treatment of fruit with 1‐MCP after harvest may suppress the climacteric production of ethylene. An increased respiration rate may be associated with fruit senescence and the development of disease during fruit storage. In this study, the respiration rate of mango showed a typical climacteric pattern under ambient storage (Figure 2b). In the 1‐MCP‐treated group, the respiration rate was significantly lower than that recorded in the control group after six days of storage (*p* < .05). In the 1‐MCP‐treated group, peak respiration was reached more slowly than in the control group, indicating that treatment with 1‐MCP reduced the respiration of mango.

**Figure 3:**
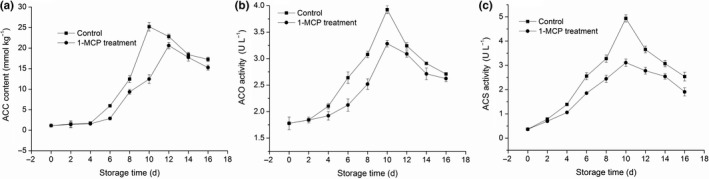
Effects of 1‐MCP on ACC content (a), ACC oxidase activity (b), and ACC synthase activity (c) in mango fruit at 25°C during sixteen days. Results represent the mean ± standard error (SE)

In both groups, the ACC content was initially increased, followed by a decrease throughout the entire storage duration (Figure 3a). Treatment with 1‐MCP significantly (*p* < .05) inhibited the increase in the ACC content on days 6, 8, and 10. In the 1‐MCP‐treated group, the ACC content peaked on day 12 of storage, two days later compared with the control group. The activity of ACO increased during the first ten days of storage and declined afterward (Figure 3b) in both groups. Treatment with 1‐MCP significantly reduced the ACO activity on days 6, 8, and 10 (all *p* < .05). The same trend was observed in the activity of ACS in both groups (Figure 3c). In the 1‐MCP‐treated group, the ACS activity was lower than that observed in the control group after four days of storage (*p* < .05). These results confirmed that treatment with 1‐MCP inhibited the production of ethylene. This effect was closely related to the ACC content and the activities of ACO and ACS.

### 3.3 Cloning and sequences of the *MiETR1* and *MiERS1* genes

**Figure 4:**
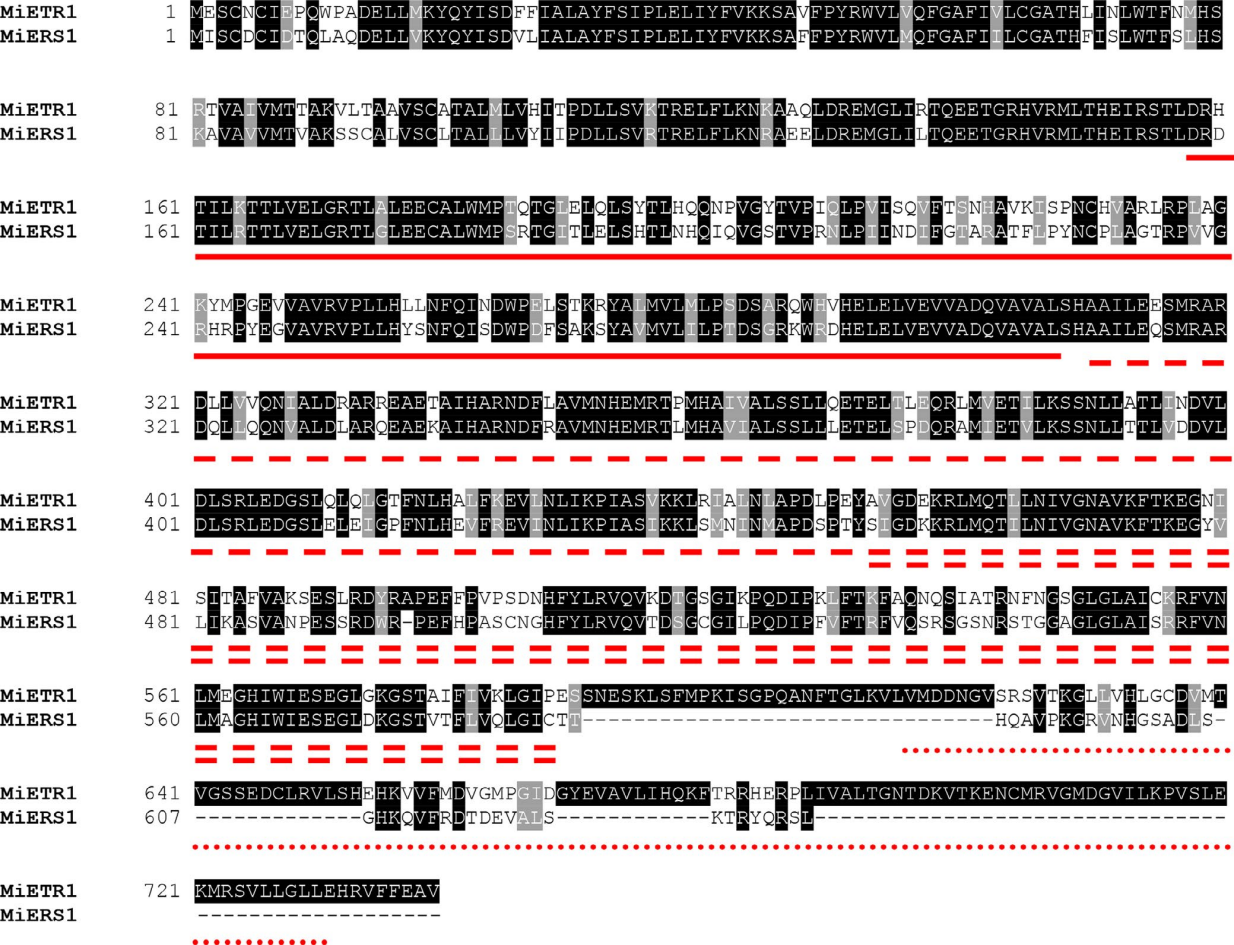
Amino acid sequence and structural features of Mangifera indica L. (Tainong 1) ETR1 and ERS1 protein. The GAF domain implicated in protein–protein interactions is marked with a solid line. His kinase domains are marked with dashed line, indicating a signal transduction histidine kinase (“－－－”) and a diverged histidine kinase‐like ATPases domain (“＝＝＝”). The receiver domain is marked with a dotted line only in MiETR1

The full‐length cDNAs of two ethylene receptor genes in mango were obtained (Figure 4). The *MiETR1* gene was 2,570 bp in length with a 113 bp 3′‐untranslated region, a poly‐A tail, and an ORF of 2,220 bp encoding 739 aa. The deduced protein had a molecular mass of 86,385 Da and a 9.09 isoelectric point. The 5′‐ and 3′‐noncoding sequences were 164 and 309 nucleotides in length, respectively. The *MiERS1* gene was 2,171 bp in length and encoded 629 aa, with a predicted molecular weight of 80,518 Da and an isoelectric point of 6.34. The 5′‐ and 3′‐noncoding sequences were 102 and 179 nucleotides in length, respectively, with a poly‐A tail and 1,890 bp ORF. The sequences of *MiETR1* and *MiERS1* were uploaded to GenBank^®^ (NCBI‐NIH) with accession numbers KY002681 and KY002682.

### 3.4 Bioinformatic analysis

**Figure 5:**
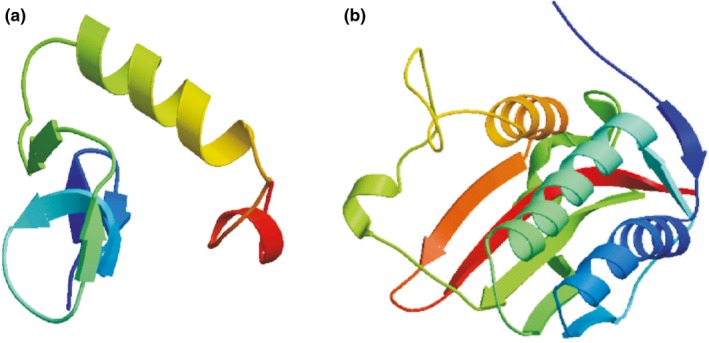
Tertiary structures of MiETR1(a) and MiERS1 (b) as predicted by 3D‐JIGSAW

A secondary structural analysis for MiETR1 and MiERS1 was carried out (://www.expasy.org). The results revealed that the putative MiETR1 peptide was composed of 24.37% alpha helix, 9.97% beta turn, and 42.93% random coil. The MiERS1 peptide consisted of 43.45% alpha helix, 7.52% beta turn, and 29.94% random coil. The major part of the secondary structure of MiETR1 was random coil, whereas the alpha helix was the major segment of the MiERS1 secondary structure. In addition, both the N‐ and C‐terminal parts were alpha helices. The tertiary structural models of MiETR1 and MiERS1 were built on the basis of the homology of proteins with known crystalline structures using protein modeling software (SWISS‐MODEL and 3D‐JIGSAW) (Figure 5). Three transmembrane regions (22–43 aa, 53–73 aa, and 82–106 aa) were identified near the N‐terminus of the MiETR1. Four transmembrane regions (22–43 aa, 53–73 aa, 82–109 aa, and 575–593 aa) were identified near the N‐terminus of MiERS1. MiETR1 did not have any coiled‐coil domains, unlike MiERS1 that contained two coiled‐coil domains (150–180 aa and 340–380 aa).

The MiERS1 and MiERS1 proteins, which contained three conserved regions (i.e., HisKA, ATPase_c, and GAF domains), belonged to the ETR1 subgroup (Figure 4). All proteins conserved the following residues: Ala31, Ile62, Cys65, Ala102, Cys4, Cys6, His354, and Asp659. Among these, Ala31, Ile62, Cys65, and Ala102 are considered to play an important role in the ordinary function of ethylene receptors (Bleecker & Schaller, 1996), the Cys4 and Cys6 residues are necessary to form the disulfide‐linked dimer (Schaller & Bleeker, 1995). Moreover, His354 and Asp659 were the presumptive sites of histidine kinase and autophosphorylation of receiver domains (Chang et al., 1993). MiERS1 and MiERS1 showed 44% homology, and the homology of the N‐terminal amino acids was higher than that observed for the C‐terminal amino acids. Moreover, MiERS1 lacked 110 aa and a signal receptor domain at the C‐terminal compared with MiERS1. The low identity observed between MiETR1 and MiERS1 may be primarily related to the lack of a receiver domain in MiERS1. The MiETR1 and MiERS1 proteins were conserved at residues deemed to be crucial for the normal function of ETR1.

**Figure 6:**
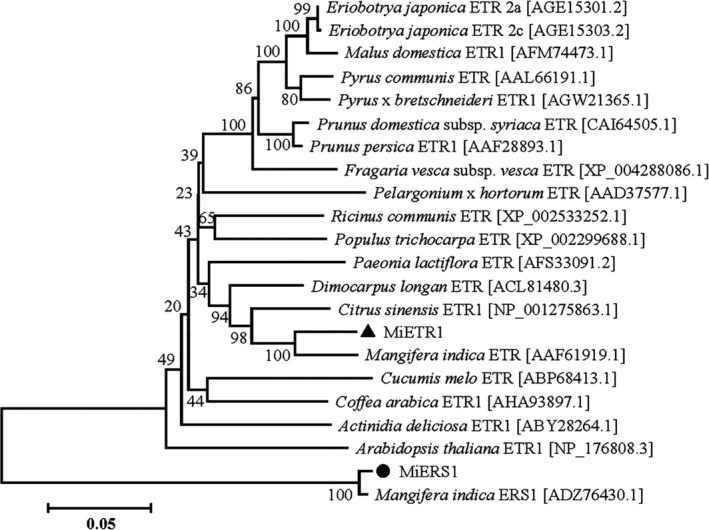
Phylogenetic analysis of MiETR1 and MiERS1 from the mango with other ETR1s from different plant species, based on alignments of amino acid sequences

A phylogenetic tree was generated using *MiETR1* and *MiERS1* sequences with other related *ETRs* and *ERSs* deposited in GenBank^®^ (NCBI‐NIH) (Figure 6). As shown in Figure 6, mango *MiETR1* shared a homology with that of other fruit. *MiETR1* shared the highest homology (91%) with *ETR*s from *Citrus sinensis* (KDO50509.1) and *Citrus clementina* (XP006442239.1). A relatively high similarity was found for DNA sequences of *ETR*s from *Arabidopsis thaliana* (NP176808.3) (85%) and *Glycine max* (XP006592983.1) (83%). *MiERS1* shared the highest homology (91%) with *ERS*s from *Citrus sinensis* (AAC99435.1) and *Citrus clementina* (XP006444412.1). A relatively high similarity was found for *ERS* DNA sequences from *Arabidopsis thaliana* (NP181626.1) (73%) and *Glycine max* (XP006604726.1) (75%).

### 3.5 Expression analysis of *MiETR1* and *MiERS1* treated with 1‐MCP

**Figure 7:**
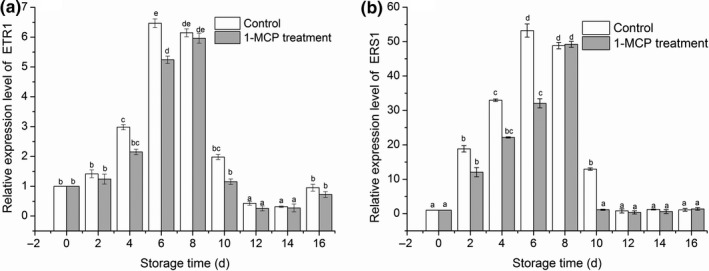
Effects of 1‐MCP treatment on the expression of MiETR1 (a) and MiERS1 (b) in mango fruit at 25°C during sixteen days. Results represent the mean ± standard error (SE). A different superscript letter in the same column indicates statistical difference (*p* < .05)

The relationship between the climacteric production of ethylene and the levels of *MiETR1* and *MiERS1* mRNA in postharvest mango was further analyzed. Real‐time PCR using gene‐specific primers was used to explore the expression patterns of *MiETR1* and *MiERS1* genes. In both groups, the expression of *MiETR1* mRNA was shown to be upregulated as the storage duration extended (Figure 7a). The expression peaked on day 6 (6.47‐fold higher levels vs. those observed in the control group on day 0), followed by a decrease on day 8. A similar pattern was recognized in an *ETR*‐type gene in muskmelon (Sato‐Nara et al., 1999) and tomato (*LeETR4* and *LeETR5*) (Tieman & Klee, 1999). Treatment of mango with 1‐MCP was linked to a reduction in the expression of *MiETR1*. A relatively higher expression of *MiETR1* mRNA was found in the control group compared with the 1‐MCP‐treated group. Treatment with 1‐MCP impeded the expression of *MiETR1* significantly on days 4, 6, and 10 (all *p* < .05). Similar to *MiETR1*, the *MiERS1* mRNA levels were found to be upregulated as the storage duration extended and reached its maximum on day 6 (53.2‐fold higher levels than those recorded in the control group on day 0) (Figure 7b). The *MiERS1* mRNA levels changed dynamically according to the different storage durations. Treatment with 1‐MCP impeded the expression of *MiETR1* significantly on days 2, 4, 6, and 10 (all *p* < .05). These results indicated that MiETR1 and MiERS1 may have the ability to combine the putative ethylene receptors with ethylene. Treatment with 1‐MCP prohibited the binding of ethylene to the ethylene receptor site and protein.

## 4 DISCUSSION

In climacteric fruit, ethylene regulates various and complex responses during growth and development, including ripening and senescence. 1‐MCP is an inhibitor of ethylene perception in plant tissues. Treatment with 1‐MCP delayed senescence in mango by inhibiting enzymatic activity (ACS and ACO) involved in ethylene biosynthesis. In the present study, treatment with 1‐MCP markedly delayed the reduction in firmness and TSS content (Figure 1). Moreover, it inhibited the rate of respiration (Figure 2b) as well as the activities of ACO and ACS (Figure 3), resulting in a reduction of the accumulation of ACC. This may also lead to reduced production of ethylene and a delayed peak of ethylene production by 2 days compared with untreated mango fruit (Figure 2a). This delayed onset of ethylene climacteric by 1‐MCP has also been reported for banana (Golding, Shearer, Wyllie, & McGlasson, 1998), avocado (Feng, Apelbaum, Sisler, & Goren, 2000), plum (Valero, Martinezromero, Valverde, Guillen, & Serrano, 2003), persimmon (Luo, 2007), pears (Lu, Cureatz, & Toivonen, 2009), and pineapples (Selvarajah, Bauchot, & John, 2001).Therefore, it is suggested that treatment of mango with 1‐MCP may prolong the storage life and improve the storage quality.

Ethylene receptors are initial factors participating in the ethylene signal transduction pathway (Shakeel et al., 2013). They are participated in numerous biological processes, negatively regulating the ethylene response and inhibiting responses under the insufficiency of ethylene with the N‐terminal domain regulating the receptor signaling condition (Li et al., 2016; Merchante, Alonso, & Stepanova, 2013; Shakeel et al., 2013). Ethylene receptor genes have been identified in many plant species, and their expression patterns have been examined (Rasori et al., 2002). In the present study, two ethylene receptor genes, namely *MiETR1* and *MiERS1*, were extracted from mango and found to be similar to those identified in the corresponding genes of *Citrus sinensis*, *Citrus clementina*, *Arabidopsis thaliana*, and *Glycine max* (Figure 4)*. MiETR1* and *MiERS1* are members of a multigene family found in numerous species (Bleecker, 1999). At the N‐terminus, the clone also includes three hydrophobic regions, with features of ETR homologs (Figure 5). The *MiETR1* and *MiERS1* genes contain a histidine kinase domain and a sensor domain, playing crucial roles in the normal function of ethylene receptors and ETR‐ or ERS‐type proteins (Bleecker, 1999). The main difference between MiETR1 and MiERS1 is the loss of a receiver response domain on MiERS1, as observed in other ETR1‐ and ERS1‐type proteins (Figure 4). Considering that the histidine kinase and response regulator domains are included in the C‐terminus of ETR1, this region may be involved in transferring the ethylene signal. Therefore, in mango, there may be two different ETR1 polypeptides that interact differently with the downstream effectors. Although the present results indicated that the biochemical properties of ETR1 and ERS1 were similar at the signal perception level, differences in their structures might lead to different signaling patterns. Loss of the response regulator domain of ERS1 was also identified in ERS2 among five members, ETR1, ERS1, ETR2, ERS2, and EIN4 of ethylene receptor family (Shakeel et al., 2013). The response regulator domain has been shown to be involved in the transfer of phosphate in other well‐characterized signal pathways (Contreras‐Vergara et al., 2012; Pang et al., 2007; Rasori et al., 2002). Hence, this difference in structure may affect signaling to downstream effectors.

The pattern of ethylene receptor mRNA expression is parallel to the pattern of fruit ripening (Ish‐Shalom, Dahan, Maayan, & Irihimovitch, 2011; Martínez et al., 2001). In this study, the mRNA levels of *MiETR1* and *MiERS1* increased dynamically during the early stages of mango fruit storage (Figure 7). The expression patterns of *MiETR1* and *MiERS1* in mango suggested that these two ethylene receptors hold important functions in ethylene signal transduction. Furthermore, these findings indicated that MiETR1 and MiERS1 might be putative ethylene receptors with an ethylene‐binding ability. Treatment with 1‐MCP suppressed the expression of *MiETR1* and *MiERS1* in mango stored at room temperature. These findings indicated that the changes in the expression of ethylene receptor genes caused by 1‐MCP treatment may contribute to the interaction between 1‐MCP and ethylene receptors. Yamamoto et al. (1995) demonstrated that the genes coding for ACO and ACS are linked to the expression of the ethylene receptor in melon fruit. In mango, a similar ripening pattern was found, implying a link between the expression of the ethylene receptor and the activities of ACO and ACS (Pathak et al., 2003). The increased expression of the ethylene receptor, along with the higher sensitivity and production of ethylene, was discovered in petioles of tomato (Syariful et al., 2019). Inhibition of ethylene by 1‐MCP is based on the ability of 1‐MCP to irreversibly bind to ethylene receptors, consequently diminishing the normal increase in ACS and ACO enzyme activities during ripening and senescence (Binder & Bleecker, 2003).

## 5 CONCLUSION

Treatment with 1‐MCP was effective in delaying the evolution of mango postharvest ripening, including firmness, TSS and TA. 1‐MCP treatment inhibited the production of ethylene in fruit, which was correlated with the ACC content and the activity of ACO and ACS. In addition, MiETR1 and MiERS1 have important functions in ethylene signal transduction. Altogether, these results indicated that treatment with 1‐MCP effectively prevented ethylene‐induced ripening and senescence by regulation of ethylene biosynthesis and ethylene receptor gene (*MiETR1* and *MiERS1*) expression in mango. This study presents an innovative method for prolonging the storage life of harvest mango through the regulation of expression. Further research is warranted to understand the roles of each member of the ETR family in the process of senescence in mango. The molecular mechanism of ethylene response to regulation through ethylene receptor isoforms in mango remains unclear. In addition, the genetic and physiological characterization of the ETR family is necessary to better understand its function and role in regulating senescence in mango.

## CONFLICT OF INTEREST

The authors notify that there are no conflicts of interest.

## ETHICAL STATEMENT

This study does not involve any human or animal testing.

## References

[fsn31417-bib-0001] Amornputti, S. , Ketsa, S. , & Doorn, W. G. V. (2016). 1‐Methylcyclopropene (1‐MCP) effects on ethylene biosynthesis in relation to flesh browning of ‘Empire’ apple fruit. Postharvest Biology & Technology, 114(20), 69–75.

[fsn31417-bib-0002] An, J. , Zhang, M. , Lu, Q. , & Zhang, Z. (2006). Effect of a prestorage treatment with 6‐benzylaminopurine and modified atmosphere packaging storage on the respiration and quality of green asparagus spears. Journal of Food Engineering, 77(4), 951–957. 10.1016/j.jfoodeng.2005.08.024

[fsn31417-bib-0003] Binder, B. M. , & Bleecker, A. B. (2003). A model for ethylene receptor function and 1‐Methylcyclopropane action. Acta Horticulturae., 628, 177–187.

[fsn31417-bib-0004] Bleecker, A. B. (1999). Ethylene perception and signalling: An evolutionary perspective. Trends in Plant Science, 4(7), 269–274. 10407443 10.1016/s1360-1385(99)01427-2

[fsn31417-bib-0005] Bleecker, A. B. , & Kende, H. (2000). Ethylene: Agaseous Signal Molecule in Plants. Annual Review of Cell and Developmental Biology, 16, 1–18. 10.1146/annurev.cellbio.16.1.1 11031228

[fsn31417-bib-0006] Bleecker, A. B. , & Schaller, G. E. (1996). The mechanism of ethylene perception. Plant Physiology, 111(3), 653–660. 10.1104/pp.111.3.653 12226320 PMC157880

[fsn31417-bib-0007] Boller, T. , Herner, R. C. , & Kende, H. (1979). Assay for an enzymatic formation of an ethylene precursor, 1‐aminocyclopropane‐1‐carboxylic acid. Planta, 145(3), 293–303. 24317737 10.1007/BF00454455

[fsn31417-bib-0008] Chang, C. , Kwok, S. F. , Bleecker, A. B. , & Meyerowitz, E. M. (1993). Arabidopsis ethylene‐response gene *ETR1*: Similarity of product to two‐component regulators. Science, 262(5133), 539–544. 10.1126/science.8211181 8211181

[fsn31417-bib-0009] Contreras‐Vergara, C. A. , Stephens‐Camacho, N. A. , Yepiz‐Plascencia, G. , González‐Aguilar, G. A. , Arvizu‐Flores, A. A. , Sanchez‐Sanchez, E. , & Islas‐Osuna, M. A. (2012). Cloning and expression of ethylene receptor ERS1 at various developmental and ripening stages of mango fruit. Genetics and Molecular Research, 11(4), 4081–4092. 10.4238/2012.September.10.6 23079970

[fsn31417-bib-0010] Feng, X. , Apelbaum, A. , Sisler, E. C. , & Goren, R. (2000). Control of ethylene responses in avocado fruit with 1‐methylcyclopropene. Postharvest Biology and Technology, 20(2), 143–150. 10.1016/S0925-5214(00)00126-5

[fsn31417-bib-0011] Golding, J. B. , Shearer, D. , Wyllie, S. G. , & McGlasson, W. B. (1998). Application of 1‐MCP and propylene to identify ethylene‐dependent ripening process in mature banana fruit. Postharvest Biology and Technology, 14(1), 87–98.

[fsn31417-bib-0012] Hua, J. , Chang, C. , Sun, Q. , & Meyerowitz, E. (1995). Ethylene insensitivity conferred by arabidopsis ers gene. Science, 269(5231), 1712–1714. 7569898 10.1126/science.7569898

[fsn31417-bib-0013] Hua, J. , Sakai, H. , Nourizadeh, S. , Chen, Q. G. , Bleecker, A. B. , & Meyerowitz, E. E. M. (1998). Ein4 and ers2 are members of the putative ethylene receptor gene family in arabidopsis. The Plant Cell, 10(8), 1321–1332. 9707532 10.1105/tpc.10.8.1321 PMC144061

[fsn31417-bib-0014] Ish‐Shalom, M. , Dahan, Y. , Maayan, I. , & Irihimovitch, V. (2011). Cloning and molecular characterization of an ethylene receptor gene, miers1, expressed during mango fruitlet abscission and fruit ripening. Plant Physiology & Biochemistry, 49(8), 931–936. 10.1016/j.plaphy.2011.05.010 21676621

[fsn31417-bib-0015] Jiang, Y. , & Fu, J. (2000). Ethylene regulation of fruit ripening: Molecular aspects. Plant Growth Regulation, 30(3), 193–200.

[fsn31417-bib-0016] Karakurt, Y. , Tonguç, M. , & Ünlü, H. Ö. (2014). The molecular characterization and expression analyses of ethylene receptor genes from watermelon fruit. Turkish Journal of Botany, 38(6), 1123–1131. 10.3906/bot-1405-29

[fsn31417-bib-0017] Kuroda, S. , Hirose, Y. , Shiraishi, M. , Davies, E. , & Abe, S. (2004). Co‐expression of an ethylene receptor gene, ers1, and ethylene signaling regulator gene, ctr1, in delphinium during abscission of florets. Plant Physiology and Biochemistry, 42(9), 745–751. 10.1016/j.plaphy.2004.07.006 15474381

[fsn31417-bib-0018] Lalel, H. J. D. , Singh, Z. , & Tan, S. C. (2003). The role of ethylene in mango fruit aromavolatiles biosynthesis. Journal of Horticultural Science and Biotechnology, 78(4), 485–496. 10.1080/14620316.2003.11511653

[fsn31417-bib-0019] Li, Y. H. , Wu, Q. S. , Huang, X. , Liu, S. H. , Zhang, H. N. , Zhang, Z. , & Sun, G. M. (2016). Molecular cloning and characterization of four genes encoding ethylene receptors associated with pineapple (*Ananas comosus* L.) flowering. Frontiers in Plant Science, 7, 710–725. 10.3389/fpls.2016.00710 27252725 PMC4878293

[fsn31417-bib-0020] Li, Z. Q. , Qiao, Y. S. , Tong, Z. G. , Zhou, J. , & Zhang, Z. (2010). Effect of ethylene and 1‐mcp on post‐harvest physiology and on expression of the ethylene receptor genes ppetr3 and ppers2 in pear (pyrus pyrifolia nakai akikusui’ikusuiia *Journal of Pomology & Horticultural* . Science, 85(1), 71–77.

[fsn31417-bib-0021] Livak, K. J. , & Schmittgen, T. D. (2001). Analysis of relative gene expression data using real‐time quantitative PCR and the 2‐ΔΔCT method. Methods, 25(4), 402–408. 10.1006/meth.2001.1262 11846609

[fsn31417-bib-0022] Lizada, M. C. , & Yang, S. F. (1979). Simple and sensitive assay for 1‐aminocyclopropane‐1‐carboxylic acid. Analytical Biochemistry, 100(1), 140–145. 543532 10.1016/0003-2697(79)90123-4

[fsn31417-bib-0023] López‐Gómez, R. , & Gómez‐Lim, M. A. (1992). A method for extracting intact RNA from fruits rich in polysaccharides using ripe mango mesocarp. HortScience, 27(5), 440–442. 10.21273/HORTSCI.27.5.440

[fsn31417-bib-0024] Lu, C. W. , Cureatz, V. , & Toivonen, P. A. (2009). Improved quality retention of packaged ‘Anjou’ pear slices using a 1‐methylcyclopropene (1‐MCP) co‐release technology. Postharvest Biology and Technology, 51(3), 378–383. 10.1016/j.postharvbio.2008.09.004

[fsn31417-bib-0025] Luo, Z. S. (2007). Effect of 1‐methylcyclopropene on ripening of postharvest persimmon (*Diospyros kaki* L.) fruit. Food Science & Technology, 40(2), 285–291. 10.1016/j.lwt.2005.10.010

[fsn31417-bib-0026] Martínez, P. G. , Gómez, R. L. , & Gómez‐Lim, M. A. (2001). Identification of an ETR1‐homologue from mango fruit expressing during fruit ripening and wounding. Journal of Plant Physiology, 158(1), 101–108. 10.1078/0176-1617-00238

[fsn31417-bib-0027] Merchante, C. , Alonso, J. M. , & Stepanova, A. N. (2013). Ethylene signaling: Simple ligand, complex regulation. Current Opinion in Plant Biology, 16(5), 554–560. 10.1016/j.pbi.2013.08.001 24012247

[fsn31417-bib-0028] Pang, J. H. , Ma, B. , Sun, H. J. , Ortiz, G. I. , Imanishi, S. , Sugaya, S. , … Ezura, H. (2007). Identification and characterization of ethylene receptor homologs expressed during fruit development and ripening in persimmon (*Diospyros * *kaki* Thumb.). Postharvest Biology and Technology, 44(3), 195–203. 10.1016/j.postharvbio.2006.12.017

[fsn31417-bib-0029] Pathak, N. , Asif, M. H. , Dhawan, P. , Srivastava, M. K. , & Nath, P. (2003). Expression and activities of ethylene biosynthesis enzymes during ripening of banana fruits and effect of 1‐MCP treatment. Plant Growth Regulation, 40(1), 11–19.

[fsn31417-bib-0030] Rasori, A. , Ruperti, B. , Bonghi, C. , Tonutti, P. , & Ramina, A. (2002). Characterization of two putative ethylene receptor genes expressed during peach fruit development and abscission. Journal of Experimental Botany, 53(379), 2333–2339. 10.1093/jxb/erf097 12432026

[fsn31417-bib-0032] Sakai, H. , Hua, J. , Chen, Q. G. , Chang, C. , Medrano, L. J. , Bleecker, A. B. , & Meyerowitz, E. M. (1998) . ETR2 is an ETR1‐like gene controlling ethylene signal in Arabidopsis. Proceedings of the National Academy of Sciences of the United States of America, 95(10), 5812–5817. 9576967 10.1073/pnas.95.10.5812 PMC20462

[fsn31417-bib-0033] Salinas‐Roca, B. , Soliva‐Fortuny, R. , Welti‐Chanes, J. , & Martín‐Belloso, O. (2018). Effect of pulsed light, edible coating, and dipping on the phenolic profile and antioxidant potential of fresh‐cut mango. Journal of Food Processing & Preservation, 42(5), e13591 10.1111/jfpp.13591

[fsn31417-bib-0034] Sato‐Nara, K. , Yuhashi, K.‐I. , Higashi, K. , Hosoya, K. , Kubota, M. , & Ezura, H. (1999). Stage‐ and tissue‐specific expression of ethylene receptor homolog genes during fruit development in muskmelon. Plant Physiology, 120(1), 321–329. 10.1104/pp.120.1.321 10318709 PMC59264

[fsn31417-bib-0035] Schaller, G. E. , & Bleecker, A. B. (1995). Ethylene‐binding sites generated in yeast expressing the arabidopsis etr1 gene. Science, 270(5243), 1809–1811. 8525372 10.1126/science.270.5243.1809

[fsn31417-bib-0036] Selvarajah, S. , Bauchot, A. D. , & John, P. (2001). Internal browning in cold‐stored pineapples is suppressed by a postharvest application of 1‐methylcyclopropene. Postharvest Biology and Technology, 23(2), 167–170. 10.1016/S0925-5214(01)00099-0

[fsn31417-bib-0037] Shakel, S. , Wang, X. , Binder, B. M. , & Schaller, G. E. (2013). Mechanisms of signal transduction by ethylene: Overlapping and non‐overlapping signalling roles in a receptor family. Annals of Botany, 5(1), plt010. 10.1093/aobpla/plt010 PMC3611092 23543258

[fsn31417-bib-0038] Sherman, A. , Rubinstein, M. , Eshed, R. , Benita, M. , Ish‐Shalom, M. , Sharabi‐Schwager, M. , … Ophir, R. (2015). Mango (*Mangifera indica* L.) germplasm diversity based on single nucleotide polymorphisms derived from the transcriptome. BMC Plant Biology, 15(1), 277–287. 26573148 10.1186/s12870-015-0663-6 PMC4647706

[fsn31417-bib-0040] Syariful, M. , Ken, H. , Yoshihiro, O. , Ryoichi, Y. , Matthew, D. , Tohru, A. , & Hiroshi, E. (2019). Evidence of the functional role of the ethylene receptor genes SlETR4 and SlETR5 in ethylene signal transduction in tomato. Molecular Genetics and Genomics, 294, 301–313. 10.1007/s00438-018-1505-7 30382349

[fsn31417-bib-0041] Tieman, D. M. , & Klee, H. J. (1999). Differential expression of two novel members of the tomato ethylene‐receptor family. Plant Physiology, 120(1), 165–172. 10.1104/pp.120.1.165 10318694 PMC59248

[fsn31417-bib-0042] Trujillo‐Moya, C. , & Gisbert, C. (2012). The influence of ethylene and ethylene modulators on shoot organogenesis in tomato. Plant Cell, Tissue and Organ Culture (PCTOC), 111(1), 41–48. 10.1007/s11240-012-0168-z

[fsn31417-bib-0043] Valero, D. , Martinezromero, D. , Valverde, J. , Guillen, F. , & Serrano, M. (2003). Quality improvement and extension of shelf life by 1‐methylcyclopropene in plum as affected by ripening stage at harvest. Innovative Food Science & Emerging Technologies, 4(3), 339–348. 10.1016/S1466-8564(03)00038-9

[fsn31417-bib-0046] Yamamoto, M. , Miki, T. , Ishiki, Y. , Fujinami, K. , Yanagisawa, Y. , Nakagawa, H. , … Sato, T. (1995). The synthesis of ethylene in melon fruit during the early stages of ripening. Plant and Cell Physiology, 36(4), 591–596.

[fsn31417-bib-0048] You, X. , Zhang, Y. , Li, L. , Li, Z. , Li, M. , Li, C. , … Sun, J. (2014). Cloning and molecular characterization of phospholipase D (*PLD*) delta gene from longan (*Dimocarpus longan* Lour.). Molecular Biology Reports, 41(7), 4351–4361. 10.1007/s11033-014-3306-3 24590739

[fsn31417-bib-0050] Zheng, Q. L. , Nakatsuka, A. , Taira, S. , & Itamura, H. (2005). Enzymatic activities and gene expression of 1‐aminocyclopropane‐1‐carboxylic acid (acc) synthase and acc oxidase in persimmon fruit. Postharvest Biology and Technology, 37(3), 286–290. 10.1016/j.postharvbio.2005.05.002

